# Proteomic analysis of HIV-1 Gag interacting partners using proximity-dependent biotinylation

**DOI:** 10.1186/s12985-015-0365-6

**Published:** 2015-09-11

**Authors:** Valerie Le Sage, Alessandro Cinti, Fernando Valiente-Echeverría, Andrew J. Mouland

**Affiliations:** HIV-1 RNA Trafficking Laboratory, Lady Davis Institute at the Jewish General Hospital, Montréal, Québec H3T 1E2 Canada; Department of Medicine, McGill University, Montréal, Québec H3A 0G4 Canada; Present address: Molecular and Cellular Virology Laboratory, Virology Program, Institute of Biomedical Sciences, Faculty of Medicine, Universidad de Chile, Independencia, 834100 Santiago, Chile

## Abstract

**Background:**

The human immunodeficiency virus type 1 (HIV-1) Gag polyprotein is necessary and sufficient to assemble non-infectious particles. Given that HIV-1 subverts many host proteins at all stages of its life cycle, it is essential to identify these interactions as potential targets for antiretroviral therapy.

**Findings:**

This work demonstrates the use of proximity-dependent biotin identification (BioID) of host proteins and complexes that are proximal to the N-terminal domains of the HIV-1 Gag polyprotein. Two of the hits identified in the BioID screen were validated by immunoprecipation and confirmed the interaction of DDX17 and RPS6 with HIV-1 Gag.

**Conclusions:**

Our results show that BioID is both a successful and complementary method to screen for nearby interacting proteins of HIV-1 Gag during the replicative cycle in different cell lines.

**Electronic supplementary material:**

The online version of this article (doi:10.1186/s12985-015-0365-6) contains supplementary material, which is available to authorized users.

## Findings

Human immunodeficiency virus type 1 (HIV-1) is the etiological agent of acquired immunodeficiency syndrome (AIDS), which specifically infects CD4+ T-cells, macrophages and dendritic cells. The integrated proviral DNA is transcribed to generate multiply and singly spliced and full-length (vRNA) messenger RNAs (mRNA). vRNA acts as both the mRNA for the Gag and Gag-Pol polyprotein precursors, and the genome that is encapsidated into assembling particles [1]. The structural polyprotein Gag mediates virus assembly by trafficking to the plasma membrane, where it multimerizes to form spherical immature virus particles that are then released [2]. During the budding process, the Gag precursor is cleaved by HIV-1 protease into mature Gag proteins p17 matrix (MA), p24 capsid (CA), p7 nucleocapsid (NC) and p6. The small size of the HIV-1 genome makes it reliant on host cellular machineries to replicate and interacts with host factors in order to neutralize host defenses and elicit pathogenesis.

### Expression of BirA*-Gag

**Fig. 1:**
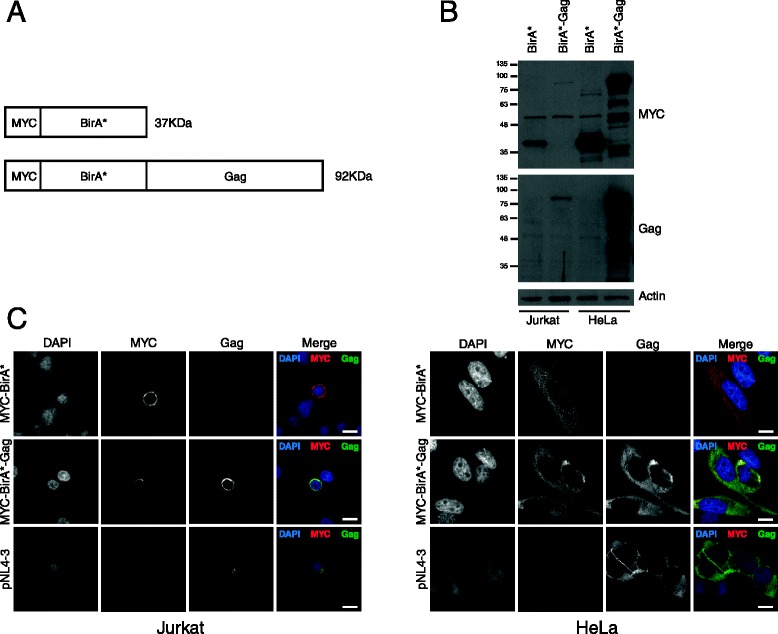
Expression and localization of recombinant HIV-1 Gag. **a** Diagram of the expression cassette encoding MYC tagged BirA* alone and MYC-BirA* fused to the N-terminus of HIV-1. **b** Jurkat or HeLa cells were transfected with BirA* or BirA*-Gag for 24 h. The expression levels of BirA* and BirA*-Gag were evaluated by Western blot using anti-MYC (US Biological Life Sciences, M9601-30), anti-p24 (NIH, 183-H12-5C) and anti-actin (Abcam, ab8226) antibodies. **c** Immunofluorescence of Jurkat (left panels) or HeLa (right panels) cells transfected with BirA*, BirA*-Gag or the HIV-1-expressing plasmid (pNL4-3). Paraformaldehyde-fixed cells were incubated with primary anti-MYC and anti-p24 antibodies for 1 h at 37 °C and secondary antibodies Alexa Fluor 488 anti-mouse IgG and Alexa Fluor 594 anti-rabbit IgG (Molecular Probes). Coverslips were mounted in ProLong Gold antifade reagent (Molecular Probes) with DAPI to stain the nuclei. Images were acquired using a Wave FX Spinning disc confocal microscope with a 63x objective. Scale bar indicates 10 μm

The structural protein Gag is sufficient by itself to produce HIV-1 virus-like particles (VLPs). VLPs cannot undergo a full replication cycle nor produce new progeny virus, which has allowed them to be applied to gene therapy, as they can enter target cells to deliver specific genes [3]. The goal of this study was to investigate potential novel host proteins that interact with the HIV-1 Gag polyprotein using a proximity-dependent biotin identification (BioID) screen [4]. This approach relied on the proximity-dependent biotinylation of nearby and interacting proteins by a promiscuous biotin ligase, BirA*, that was fused to the N-terminus of HIV-1 Gag. The biotinylated proteins were then affinity purified and identified by LC-tandem mass spectrometry (MS). To this end, we cloned HIV-1 Gag into pcDNA3.1 mycBioID (Addgene plasmid 35700) [4] to generate BirA*-Gag (Fig. 1a). Jurkat (T lymphocyte) and HeLa cells were transiently transfected with BirA* or BirA*-Gag and protein expression levels were determined by Western blot analysis (Fig. 1b). As shown in Fig. 1b, as a result of a lower transfection efficiency, Jurkat cells expressed the BirA*-Gag at a much lower level than did the HeLa cells. Immunofluorescence demonstrated that there was extensive colocalization of the MYC and p24 fluorescence in BirA*-Gag-expressing cells (Fig. 1c, middle panels). The BirA*-Gag fusion protein was localized appropriately in the cytoplasm and to the plasma membrane (Fig. 1c, bottom panels), as compared to cells transfected with the HIV-1-expressing plasmid pNL4-3 (Fig. 1c, bottom panels).

### Identification of potential cellular interacting partners of BirA*-Gag

**Fig. 2:**
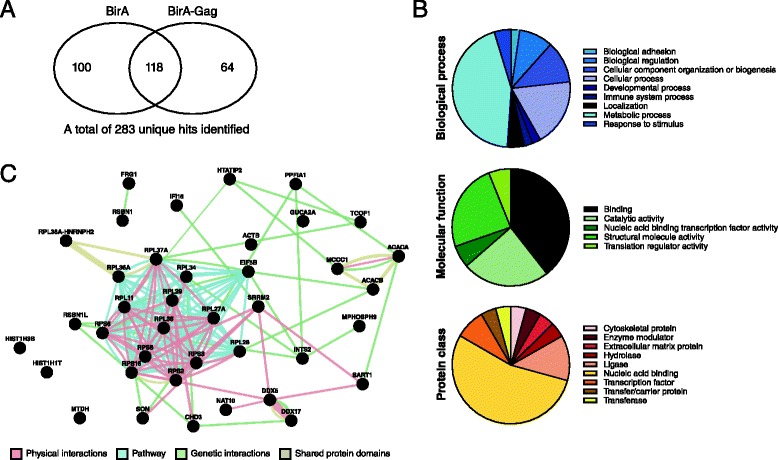
Identification of potential Gag proximal and interacting partners. Jurkat cells were transfected with BirA* or BirA*-Gag in medium supplemented with 50 μM Biotin (Sigma). Cell lysates were harvested 24 h post-transfection. Dynabeads MyOne Streptavidin C1 (Life Technologies) were used to precipitate biotinylated proteins from 1 mg of lysate as described by the manufacturer. The eluted proteins were separated on 4-15 % SDS-PAGE (Bio-Rad), the bands excised and analyzed by LC-tandem MS at SAMS Centre for proteomics, University of Calgary. **a** Venn diagram depicting the number of overlapping hits between Gag-interacting proteins. **b** Genes families and subfamilies were grouped and classified, using PANTHER, according to their function in three different ways: biological process, molecular function and protein class. **c** GeneMANIA (used in automatically selected weighting method) was used to determine known interactions between input genes and how they interact. The relationships between genes in the network included physical and genetic interactions, pathways and protein domain similarity

To identify proximal interactors for HIV-1 Gag, we transfected Jurkat cells with BirA*-Gag or BirA* alone in medium supplemented with 50 μM Biotin (Sigma). Precipitation and LC-tandem MS identification of biotinylated proteins revealed 64 unique peptide hits that represent 47 proteins (Fig. 2a and Additional file 1: Table S1). Data were subjected to ontology analysis using PANTHER (http://www.pantherdb.org/) [5, 6]. In biological process, the most clusters identified were: metabolic process, cellular process and cellular component organization or biogenesis; whereas binding, catalytic activity and structural molecule activity dominated the clusters in molecular function (Fig. 2b). The protein classes related to nucleic acid binding were the most enriched with transferase, transfer/carrier protein and extracellular matrix protein exclusively found in BirA*-Gag (Fig. 2b). Physical and genetic interactions, pathways, and protein domain similarity between the genes linked with BirA*-Gag were mapped using GeneMANIA (http://www.genemania.org) [7] (Fig. 2c). Compared to the distribution of GO terms across the whole human proteome, the largest set of significantly enriched (p-value 3.16E-06) in both BirA* and BirA*-Gag groups were mRNA-binding proteins that participate in ribosomal structure. This may reflect that many RNA-binding proteins were physically close to BirA*during its translation. However, a number of these factors were exclusive to the BirA*-Gag hits, including the ribosomal protein (RP) S2, S3, S5, S6, L11, S16, L27, L28, L29 and L35, which have previously been shown to interact with different HIV-1 proteins (Additional file 1: Table S1). HIV-1 MA domain of Gag has been previously shown to interact with proteins involved in translation such as the putative histidyl-tRNA synthetase HO3, and the GTPase eIF5B [8, 9]. eIF5B, identified in Additional file 1: Table S1, has also been previously shown to interact with HIV-1 Gag [9, 10] and this association has the potential to regulate translation [9]. Moreover, picornaviruses have been shown to inhibit host cell translation early in infection in part by cleavage of eIF5B [11].

The host’s first line of defense against viral infection is the innate immune system whose activation leads to the production of proinflammatory cytokines and type I interferon (IFN) responses. The only protein classified within the GO immune system process was the IFN-inducible protein 16 (IFI16). Several classes of nucleic acid sensors have been implicated in HIV-1 RNA and DNA recognition [12] and IFI16 has been shown to colocalize and associated with lentiviral DNA in the cytoplasm of macrophages [13]. Additionally, HIV-1 disease progression was affected by the genetic variation in IFI16 with a particular single-nucleotide polymorphism in its promoter region being associated with higher CD4+ T cell counts [14]. A potential interaction between IFI16 and Gag may suggest a HIV-1 countermeasure to this important host defense.

Identified in Additional file 1: Table S1, Lyric (also known as Metadherin or astrocyte-elevated gene 1 (AEG-1)) was first identified as a HIV-1 inducible gene and has been suggested to play a role in a positive-feedback loop promoting HIV-1 replication [15] and has also been implicated in HIV-associated neuropathy [16, 15]. Engeland and colleagues recently demonstrated that Lyric interacts specifically with Gag and is incorporated into HIV-1 virions, where it is cleaved by the viral protease [17].

### Validation of BirA*-Gag interactions

**Fig. 3:**
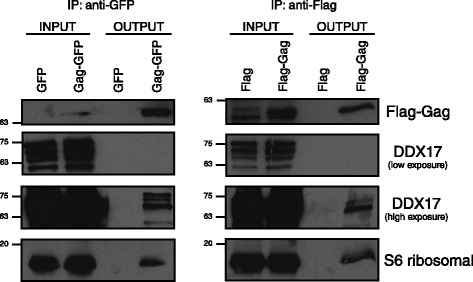
Validation of BirA*-Gag interactions by immunoprecipitation and Western blot. The Gag coding sequence was cloned into the pCI-Neo-Flag (Flag) (Life Technologies) between *XhoI* and *NotI* to express Flag-Gag. HeLa cells were transfected with either GFP or Gag-GFP (left panels) or Flag or Flag-Gag (right panel) for 24 h. Cell lysates were washed with PBS and collected using NP40 lysis buffer (50 mM Tris pH 7.8; 150 mM NaCl; 0.5 mM EDTA; 0.5 % Nonidet P-40) supplemented with Complete protease inhibitor (Roche). Cell lysates were quantified by the Bradford assay (Bio-Rad) and 1 mg of protein was immunoprecipitated with anti-MYC tag mAb-Magnetic beads for 2 h as described by the manufacturer (MLB). The bound complexes were analyzed by SDS-PAGE using anti-p24 (NIH, 183-H12-5C), anti-DDX17 (Abcam, ab24601) and anti-S6 Ribosomal Protein (Cell Signalling 54D2) antibodies

Selected cellular targets identified by LC-tandem MS (DEAD-box RNA helicase 17 (DDX17) and RPS6) as potential interactors of BirA*-Gag were then investigated to validate the results of the screen. Co-immunoprecipitations were performed using antisera against GFP and Flag following transfection of HeLa cells with GFP or Gag-GFP [18] and Flag or Flag-Gag, respectively. Endogenous DDX17 and RPS6 were readily detected in the eluate from Gag-GFP—and Flag-Gag-expressing cells but was completely absent in the eluate from GFP—or Flag-expressing cells (Fig. 3). Similar results were obtained following a MYC immunoprecipitation using the BirA* and BirA*-Gag constructs shown in Fig. 1a (data not shown). DDX17 can form heterodimers with its paralog DDX5 [19] and has been shown to be important for the production of HIV-1 particles and HIV-1 RNA nuclear export [20, 21]. As a downstream substrate of the translational activator S6K1, RPS6 is one of the key components of the protein synthesis machinery and was shown to associate with Staufen1 ribonucleoprotein complexes isolated from HIV-1-expressing cells [22]. RPS6 is a key component of the host protein synthesis machinery and is important in modulating the overall translational capacity of cells. Different antiretroviral drugs have been shown to decrease phosphorylation of RPS6 in myocytes and negatively affect protein synthesis in these cells [23].

Recently, Ritchie and colleagues published a study in which they examined the interactions of Gag versus a MA-deleted Gag mutant in virus-producing HEK 293 T cells using BioID [24]. Fifty cellular proteins were biotinylated by wild-type Gag, however, only one, Lyric overlapped with our list (Additional file 1: Table S1). The BioID approach is very useful for identifying weak and/or transient interactions (because biotinylation occurs before solubilization) and is amenable to temporal regulation. However, limitations include the neighbouring proteins having available primary amines to be biotinylated and consequently, the absence of biotinylation does rule out interaction or proximity [4]. Our study taken together with the one performed by Ritchie and colleagues highlight the importance of the chosen location of the BirA* tag in the design of a construct, as their construct encoded BirA* within Gag between the MA and CA domains [24]. The practical labeling radius of BirA* may be as small as 10 nm [25] and therefore insertion of BirA* adjacent to or within various Gag domains will affect the pool of biotinylated interacting proteins. Another limitation may be the choice of control pulldown, expressing BirA* alone may have inadvertently eliminated, in addition to non-specific interactors, some specific Gag interactors that also interact with BirA*. This short report thoroughly validated BioID as a rapid and reliable method of screening for both proximal and interacting proteins.

## Additional file

Additional file 1:
**Proteins exclusively biotinylated by BirA*-Gag with known HIV-1 interactions.** (PDF 96 kb)

## Abbreviations

HIV-1: Human immunodeficiency virus type 1
BioID: Proximity-dependent biotin identification
AIDS: Acquired immunodeficiency syndrome
vRNA: Viral RNA
mRNA: Messenger RNA
MA: Matrix
CA: Capsid
NC: Nucleocapsid
VLPs: Virus-like particles
MS: Mass spectrometry
RP: Ribosomal protein
IFN: Interferon, IFI16, IFN-inducible protein 16
AEG-1: Astrocyte-elevated gene 1
DDX17: DEAD-box RNA helicase 17
