# Supplementary material for: Proteomic analysis of HIV-1 Gag interacting partners using proximity-dependent biotinylation
Source: Virol J. 2015 Sep 11;12:138. doi: 10.1186/s12985-015-0365-6 (PMC4566291; doi:10.1186/s12985-015-0365-6)
Supplement: Additional file 1: — Proteins exclusively biotinylated by BirA*-Gag with known HIV-1 interactions. (PDF 96 kb) [file 12985_2015_365_MOESM1_ESM.pdf]

Supplemental Table 1: Proteins exclusively biotinylated by BirA*-Gag with known HIV-1 interactions

NCBI Accenssion	Protein Name	Gene Symbol	Implicated HIV-1 proteins (reference)
NP_005137.1	Squamous cell carcinoma antigen recognized by T cells	SART1	N/A [1]; Protease [2]
AAH17921.1	FSHD region gene 1 pseudogene	LOC283788	None
AAH14977.1	Metadherin (LYRIC)	MTDH	gp120 [3]; Gag [4-6]; Tat [7]; Vpr [8]
AAH20205.1<br>BAA91800.1	N-acetyltransferase 10 (GCN5-related)	NAT10	Gag [4]; Rev [9]
EAW47709.1	Ribosomal protein L27a pseudogene 6	RPL27AP6	None
EAW51024.1<br>EAW64492.1<br>AAH71674.1	Ribosomal protein S16	RPS16	Gag [10]
EAW51434.1	Integrator complex subunit 2	INTS2	None
EAW52801.1<br>AAA58683.1	Interferon, gamma-inducible protein 16	IFI16	Tat [11]; N/A [12-15]
EAW54849.1	M-phase phosphoprotein 9	MPHOSPH9	None
EAW55529.1	Histone cluster 1	HIST1H1T	Gag [16, 10]
EAW58635.1<br>AAA60288.1	Ribosomal protein S6	RPS6	N/A [1]; Rev [9]
EAW61735.1<br>EAW61737.1<br>BAG59473.1<br>BAG61214.1<br>AAH27252.1<br>CAB61367.2<br>BAD92554.1	Treacher Collins-Franceschetti syndrome 1	TCOF1	None
EAW68340.1	HIV-1 Tat interactive protein 2	HTATIP2	None
EAW68442.1	Ribosomal protein S2 pseudogene 40	RPS2P40	None
EAW70570.1	Ribosomal protein L37a	RPL37A	None
EAW75952.1	Ribosomal protein L27a	RPL27A	Gag [10]
EAW76708.1	hCG40355, isoform CRA_a	N/A	None
EAW90750.1	hCG1643303, isoform CRA_a, partial	N/A	None
EAW95067.1	Ribosomal protein L11	RPL11	Vpr [8]; Gag [10]
EAX02861.1	Ribosomal protein L36a	RPL36A	None
AAK07692.1	SON DNA binding protein	SON	None
NP_002943.2<br>BAB93525.1<br>AAH32129.1<br>AAH04520.2	Ribosomal protein S2	RPS2	N/A [1]; Rev [9]; Tat [17]
AAI50502.1	Ribosomal protein S3	RPS3	N/A [1]; Gag [10]
1WH9_A	Solution structure of the KH domain of human ribosomal protein S3	N/A	None
NP_000986.2	Ribosomal protein L34	RPL34	None
AAA35915.1	Guanylate cyclase activator 2A (guanylin)	GUCA2A	None
BAG60103.1	DEAD (Asp-Glu-Ala-Asp) box helicase 5	DDX5	N/A [1, 18]; Protease [2]; Vpr [8]; Rev [9, 19, 20]
AAH26155.1<br>BAA92075.1	Round spermatid basic protein 1	RSBN1	None
NP_001129606.1	Ribosomal protein L28	RPL28	Gag [10]
BAH14799.1<br>BAD92974.1	Methylcrotonoyl-CoA carboxylase 1 (alpha)	MCCC1	None
3FF6_A	Chain A, Human Acc2 Ct Domain With Cp-640186	ACACB	None
AAB87383.1	Chromodomain helicase DNA binding protein 3	CHD3	None
CAA45026.1	Actin, beta	ACTB	Env [21, 22]; Gag [23-25]; Nef [26, 27]; Rev [28, 29]; Tat [30]; Protease [31, 2, 32]; RT [33, 34]
NP_001186902.1	RPL36A-HNRNPH2 readthrough	RPL36A-	None

		HNRNPH2	
AAP94114.1	Acetyl-CoA carboxylase alpha	ACACA	Tat [35, 36]
XP_002343932	Protein FRG1-like isoform 1	LOC100289097	None
BAC85872.1	Round spermatid basic protein 1-like	RSBN1L	None
NP_006377.2	DEAD (Asp-Glu-Ala-Asp) box helicase 17	DDX17	N/A [1, 18]; Protease [2]; Rev [9, 19]; Gag [37, 10]
AAA52651.1	Histone cluster 1, H3b	HIST1H3B	Tat [38-41]
AAD16006.1	Eukaryotic translation initiation factor 5B	EIF5B	MA and Gag [10, 42]
NP_003617.1	Protein tyrosine phosphatase interacting protein (liprin), alpha 1	PPFIA1	None
NP_000981.1	Ribosomal protein L27a	RPL27A	Gag [10]
NP_004468.1	FSHD region gene 1	FRG1	Tat [17]
BAA83713.1 BAA83717.1	serine/arginine repetitive matrix 2	SRRM2	Tat [43]; N/A [44]
NP_009140.1	ribosomal protein L35	RPL35	Protease [2]; Gag [10]
BAD96826.1	ribosomal protein S5	RPS5	Rev [9]
CAA89008.1	ribosomal protein L29	RPL29	N/A [1]; Vpr [8]; Gag [10]

Protein specifically biotinylated by BirA*-Gag are indicated with NCBI accession number, protein name and gene symbol. The rightmost column lists references in which the protein has been identified as interacting with the indicated HIV-1 viral protein or implicated part of the virus life cycle. None, no previous reference; N/A, not applicable.

References

1. Milev MP, Ravichandran M, Khan MF, Schriemer DC, Mouland AJ. Characterization of staufen1 ribonucleoproteins by mass spectrometry and biochemical analyses reveal the presence of diverse host proteins associated with human immunodeficiency virus type 1. Frontiers in microbiology. 2012;3:367. doi:10.3389/fmicb.2012.00367.
2. Impens F, Timmerman E, Staes A, Moens K, Arien KK, Verhasselt B et al. A catalogue of putative HIV-1 protease host cell substrates. Biological chemistry. 2012;393(9):915-31. doi:10.1515/hsz-2012-0168.
3. Kang DC, Su ZZ, Sarkar D, Emdad L, Volsky DJ, Fisher PB. Cloning and characterization of HIV-1-inducible astrocyte elevated gene-1, AEG-1. Gene. 2005;353(1):8-15. doi:10.1016/j.gene.2005.04.006.
4. Engeland CE, Brown NP, Borner K, Schumann M, Krause E, Kaderali L et al. Proteome analysis of the HIV-1 Gag interactome. Virology. 2014;460-461:194-206. doi:10.1016/j.virol.2014.04.038.
5. Engeland CE, Oberwinkler H, Schumann M, Krause E, Muller GA, Krausslich HG. The cellular protein lyric interacts with HIV-1 Gag. Journal of virology. 2011;85(24):13322-32. doi:10.1128/JVI.00174-11.
6. Ghanam RH, Samal AB, Fernandez TF, Saad JS. Role of the HIV-1 Matrix Protein in Gag Intracellular Trafficking and Targeting to the Plasma Membrane for Virus Assembly. Frontiers in microbiology. 2012;3:55. doi:10.3389/fmicb.2012.00055.
7. Vartak-Sharma N, Gelman BB, Joshi C, Borgamann K, Ghorpade A. Astrocyte elevated gene-1 is a novel modulator of HIV-1-associated neuroinflammation via regulation of nuclear factor-kappaB signaling and excitatory amino acid transporter-2 repression. The Journal of biological chemistry. 2014;289(28):19599-612. doi:10.1074/jbc.M114.567644.
8. Barrero CA, Datta PK, Sen S, Deshmane S, Amini S, Khalili K et al. HIV-1 Vpr modulates macrophage metabolic pathways: a SILAC-based quantitative analysis. PloS one. 2013;8(7):e68376. doi:10.1371/journal.pone.0068376.
9. Naji S, Ambrus G, Cimermancic P, Reyes JR, Johnson JR, Filbrandt R et al. Host cell interactome of HIV-1 Rev includes RNA helicases involved in multiple facets of virus production. Molecular & cellular proteomics : MCP. 2012;11(4):M111 015313. doi:10.1074/mcp.M111.015313.
10. Valiente-Echeverria F, Melnychuk L, Vyboh K, Ajamian L, Gallouzi IE, Bernard N et al. eEF2 and Ras-GAP SH3 domain-binding protein (G3BP1) modulate stress granule assembly during HIV-1 infection. Nature communications. 2014;5:4819. doi:10.1038/ncomms5819.
11. Izmailova E, Bertley FM, Huang Q, Makori N, Miller CJ, Young RA et al. HIV-1 Tat reprograms immature dendritic cells to express chemoattractants for activated T cells and macrophages. Nature medicine. 2003;9(2):191-7. doi:10.1038/nm822.
12. Booiman T, Kootstra NA. Polymorphism in IFI16 affects CD4(+) T-cell counts in HIV-1 infection. International journal of immunogenetics. 2014;41(6):518-20. doi:10.1111/iji.12157.
13. Jakobsen MR, Bak RO, Andersen A, Berg RK, Jensen SB, Tengchuan J et al. IFI16 senses DNA forms of the lentiviral replication cycle and controls HIV-1 replication. Proceedings of the National Academy of Sciences of the United States of America. 2013;110(48):E4571-80. doi:10.1073/pnas.1311669110.

14. Monroe KM, Yang Z, Johnson JR, Geng X, Doitsh G, Krogan NJ et al. IFI16 DNA sensor is required for death of lymphoid CD4 T cells abortively infected with HIV. Science. 2014;343(6169):428-32. doi:10.1126/science.1243640.
15. Nissen SK, Hojen JF, Andersen KL, Kofod-Olsen E, Berg RK, Paludan SR et al. Innate DNA sensing is impaired in HIV patients and IFI16 expression correlates with chronic immune activation. Clinical and experimental immunology. 2014;177(1):295-309. doi:10.1111/cei.12317.
16. Gao W, Li M, Zhang J. Tandem immunoprecipitation approach to identify HIV-1 Gag associated host factors. Journal of virological methods. 2014;203:116-9. doi:10.1016/j.jviromet.2014.03.017.
17. Jarboui MA, Bidoia C, Woods E, Roe B, Wynne K, Elia G et al. Nucleolar protein trafficking in response to HIV-1 Tat: rewiring the nucleolus. PloS one. 2012;7(11):e48702. doi:10.1371/journal.pone.0048702.
18. Williams CA, Abbink TE, Jeang KT, Lever AM. Identification of RNA helicases in human immunodeficiency virus 1 (HIV-1) replication - a targeted small interfering RNA library screen using pseudotyped and WT HIV-1. The Journal of general virology. 2015;96(Pt 6):1484-9. doi:10.1099/vir.0.000092.
19. Yasuda-Inoue M, Kuroki M, Ariumi Y. Distinct DDX DEAD-box RNA helicases cooperate to modulate the HIV-1 Rev function. Biochemical and biophysical research communications. 2013;434(4):803-8. doi:10.1016/j.bbrc.2013.04.016.
20. Zhou X, Luo J, Mills L, Wu S, Pan T, Geng G et al. DDX5 facilitates HIV-1 replication as a cellular co-factor of Rev. PloS one. 2013;8(5):e65040. doi:10.1371/journal.pone.0065040.
21. Jolly C, Kashefi K, Hollinshead M, Sattentau QJ. HIV-1 cell to cell transfer across an Env-induced, actin-dependent synapse. The Journal of experimental medicine. 2004;199(2):283-93. doi:10.1084/jem.20030648.
22. Jolly C, Mitar I, Sattentau QJ. Requirement for an intact T-cell actin and tubulin cytoskeleton for efficient assembly and spread of human immunodeficiency virus type 1. Journal of virology. 2007;81(11):5547-60. doi:10.1128/JVI.01469-06.
23. Liu B, Dai R, Tian CJ, Dawson L, Gorelick R, Yu XF. Interaction of the human immunodeficiency virus type 1 nucleocapsid with actin. Journal of virology. 1999;73(4):2901-8.
24. Rey O, Canon J, Krogstad P. HIV-1 Gag protein associates with F-actin present in microfilaments. Virology. 1996;220(2):530-4. doi:10.1006/viro.1996.0343.
25. Wilk T, Gowen B, Fuller SD. Actin associates with the nucleocapsid domain of the human immunodeficiency virus Gag polyprotein. Journal of virology. 1999;73(3):1931-40.
26. Fackler OT, Kienzle N, Kremmer E, Boese A, Schramm B, Klimkait T et al. Association of human immunodeficiency virus Nef protein with actin is myristoylation dependent and influences its subcellular localization. European journal of biochemistry / FEBS. 1997;247(3):843-51.
27. Tan R, Patni H, Tandon P, Luan L, Sharma B, Salhan D et al. Nef interaction with actin compromises human podocyte actin cytoskeletal integrity. Experimental and molecular pathology. 2013;94(1):51-7. doi:10.1016/j.yexmp.2012.06.001.
28. Hofmann W, Reichart B, Ewald A, Muller E, Schmitt I, Stauber RH et al. Cofactor requirements for nuclear export of Rev response element (RRE)- and constitutive transport element (CTE)-containing retroviral RNAs. An unexpected role for actin. The Journal of cell biology. 2001;152(5):895-910.
29. Kimura T, Hashimoto I, Nishikawa M, Fujisawa JI. A role for Rev in the association of HIV-1 gag mRNA with cytoskeletal beta-actin and viral protein expression. Biochimie. 1996;78(11-12):1075-80.
30. Wu RF, Gu Y, Xu YC, Mitola S, Bussolino F, Terada LS. Human immunodeficiency virus type 1 Tat regulates endothelial cell actin cytoskeletal dynamics through PAK1 activation and oxidant production. Journal of virology. 2004;78(2):779-89.
31. Adams LD, Tomasselli AG, Robbins P, Moss B, Heinrikson RL. HIV-1 protease cleaves actin during acute infection of human T-lymphocytes. AIDS research and human retroviruses. 1992;8(2):291-5.
32. Shoeman RL, Kesselmier C, Mothes E, Honer B, Traub P. Non-viral cellular substrates for human immunodeficiency virus type 1 protease. FEBS letters. 1991;278(2):199-203.
33. Bukrinskaya A, Brichacek B, Mann A, Stevenson M. Establishment of a functional human immunodeficiency virus type 1 (HIV-1) reverse transcription complex involves the cytoskeleton. The Journal of experimental medicine. 1998;188(11):2113-25.
34. Hottiger M, Gramatikoff K, Georgiev O, Chaponnier C, Schaffner W, Hubscher U. The large subunit of HIV-1 reverse transcriptase interacts with beta-actin. Nucleic acids research. 1995;23(5):736-41.
35. Zhang HS, Ruan Z, Sang WW. HDAC1/NFkappaB pathway is involved in curcumin inhibiting of Tat-mediated long terminal repeat transactivation. Journal of cellular physiology. 2011;226(12):3385-91. doi:10.1002/jcp.22691.
36. Zhang HS, Wu MR. SIRT1 regulates Tat-induced HIV-1 transactivation through activating AMP-activated protein kinase. Virus research. 2009;146(1-2):51-7. doi:10.1016/j.virusres.2009.08.005.
37. Lorgeoux RP, Pan Q, Le Duff Y, Liang C. DDX17 promotes the production of infectious HIV-1 particles through modulating viral RNA packaging and translation frameshift. Virology. 2013;443(2):384-92. doi:10.1016/j.virol.2013.05.026.

38. Deng L, de la Fuente C, Fu P, Wang L, Donnelly R, Wade JD et al. Acetylation of HIV-1 Tat by CBP/P300 increases transcription of integrated HIV-1 genome and enhances binding to core histones. Virology. 2000;277(2):278-95. doi:10.1006/viro.2000.0593.
39. Deng L, Wang D, de la Fuente C, Wang L, Li H, Lee CG et al. Enhancement of the p300 HAT activity by HIV-1 Tat on chromatin DNA. Virology. 2001;289(2):312-26. doi:10.1006/viro.2001.1129.
40. El Kharroubi A, Piras G, Zensen R, Martin MA. Transcriptional activation of the integrated chromatin-associated human immunodeficiency virus type 1 promoter. Molecular and cellular biology. 1998;18(5):2535-44.
41. Lusic M, Marcello A, Cereseto A, Giacca M. Regulation of HIV-1 gene expression by histone acetylation and factor recruitment at the LTR promoter. The EMBO journal. 2003;22(24):6550-61. doi:10.1093/emboj/cdg631.
42. Wilson SA, Sieiro-Vazquez C, Edwards NJ, Iourin O, Byles ED, Kotsopoulou E et al. Cloning and characterization of hIF2, a human homologue of bacterial translation initiation factor 2, and its interaction with HIV-1 matrix. The Biochemical journal. 1999;342 (Pt 1):97-103.
43. Jager S, Cimermancic P, Gulbahce N, Johnson JR, McGovern KE, Clarke SC et al. Global landscape of HIV-human protein complexes. Nature. 2012;481(7381):365-70. doi:10.1038/nature10719.
44. Wojcechowskyj JA, Didigu CA, Lee JY, Parrish NF, Sinha R, Hahn BH et al. Quantitative phosphoproteomics reveals extensive cellular reprogramming during HIV-1 entry. Cell host & microbe. 2013;13(5):613-23. doi:10.1016/j.chom.2013.04.011.
